# Supplementary material for: Content-rich biological network constructed by mining PubMed abstracts
Source: BMC Bioinformatics. 2004 Oct 8;5:147. doi: 10.1186/1471-2105-5-147 (PMC528731; doi:10.1186/1471-2105-5-147)
Supplement: Additional File 2 — The original results of the above study (non-essential files are deleted to keep the file size under the limit set by BMC bioinformatics). [file 1471-2105-5-147-S2.bz2 › chilibotAdditionalFile2/dip10/505ID10880444E10672/html/ACVR2_BMP2.html]

 


 **ACVR2** and **BMP2** 
  
Found 20 abstracts in PubMed, retrieved 10.  
 

 What does Google say? 
 PDF only 
| .edu only 

---

**Interactive relationship** (e.g. stimulation, inhibition, etc)

**Neutral relationship**- BMP receptors IA, IB, and II and activin receptor  [ **ACVR2** ]  I were found to be expressed in myocytes, and BMP 2  [ **BMP2** ]  phosphorylated Smad1 and p38 MAPK.  Ref: 11408477 J Biol Chem, 2001
- METHODS To identify amino acid side chains involved in receptor binding, a collection of in vitro mutagenized human BMP 2  [ **BMP2** ]  variants was prepared and subjected to interaction analyses with use of the receptor ectodomains of BMPR IA, BMPR II, and ActR II  [ **ACVR2** ]  immobilized on a biosensor system.  Ref: 11263668 J Bone Joint Surg Am, 2001
- Activin receptor  [ **ACVR2** ]  like kinase ALK 1, ALK 2, ALK 3, and ALK 6 were able to reproduce the hypertrophic maturation of chondrocytes induced by BMP 2  [ **BMP2** ] .  Ref: 12082094 J Biol Chem, 2002

**Non-interactive relationship** (e.g. studied together, co-existance, homology, etc.)

- This capacity was positively associated with the expression of fibroblast growth factor receptor 3, bone morphogenetic protein 2  [ **BMP2** ] , and alpha1 II collagen COL2A1, and its loss was marked by the up regulation of activin receptor  [ **ACVR2** ]  like kinase 1 messenger RNA.  Ref: 11465712 Arthritis Rheum, 2001
